# Primary synovial chondromatosis of the ankle: A case report with radiologic and imaging findings

**DOI:** 10.1016/j.radcr.2024.12.007

**Published:** 2024-12-31

**Authors:** Morteza Gholipour, Mohsen Salimi, Alireza Motamedi, Fatemeh Abbasi, Bahar Behnam, Seyyedeh Reyhaneh Sadr

**Affiliations:** a Clinical Research Development Unit of Akhtar Hospital, Shahid Beheshti University of Medical Science, Tehran, Iran; b Student Research Committee, School of Medicine, Shiraz University of Medical Sciences, Shiraz, Iran; c Student Research Committee, Faculty of Medicine, Tabriz University of Medical Sciences, Tabriz, Iran; d Student Research Committee, Faculty of Medicine, Mazandaran University of Medical Sciences, Mazandaran, Iran

**Keywords:** Synovial chondromatosis, Ankle, Cartilage nodules, Case report

## Abstract

Synovial chondromatosis (SC) is a rare, benign joint disorder characterized by cartilaginous nodule formation within the synovial membrane. While SC typically affects larger joints such as the knee and hip, ankle involvement is exceptionally uncommon, with only a few cases documented in medical literature. We present a case of a 38-year-old male who experienced a rare presentation of recurrent ankle sprains and a palpable mass, ultimately diagnosed with SC in the ankle. Initial arthroscopic treatment proved insufficient due to the extensive loose bodies within the joint, necessitating a follow-up open arthrotomy to ensure complete excision. Postoperative recovery was favorable, with no recurrence observed at follow-up. This case underscores the importance of considering SC in patients with unexplained joint instability and mechanical symptoms, even in atypical joints like the ankle. Surgical intervention, particularly open arthrotomy, remains crucial for achieving effective management in advanced cases and preventing recurrence. This case report contributes to the limited literature on ankle SC and highlights the need for awareness of this rare presentation to facilitate early diagnosis and intervention.

## Introduction

Synovial chondromatosis (SC) is an uncommon joint disorder define by the development of cartilage nodules across the synovial membrane, impairing the normal function of synovial components [1,2]

The nodules can separate, forming loose bodies within the joint, and may eventually may later develop secondary calcification [3]

SC is most common in men in their 30s and 40s, typically affecting larger joints like the knee and hip, while involvement of the ankle is exceedingly rare [3,4].

SC appears in 2 types: the rare primary type, also referred to as Reichel's syndrome, and the secondary kind, which arises from trauma or chronic joint disorders [5].

Although SC is generally a benign and monoarticular condition, rare instances of malignant transformation and progression to polyarticular involvement have been documented in the literature [[6], [7], [8]].

The number of loose bodies in synovial chondromatosis can vary significantly, ranging from as few as 1 or 2 in the temporomandibular joint (TMJ) to over 120 in the ankle [9].

Patients with SC may experience no symptoms at all or may present with pain, swelling, and limited range of motion [10].

The pathophysiology of synovial chondromatosis is not yet fully understood, and despite its male sex-linked prevalence, no specific genetic link has been identified [11,12].

The management of synovial chondromatosis typically require surgical intervention, mostly through open removal of loose bodies along with synovectomy [13]. Nonetheless, for ankle involvement, arthroscopy is now considered as the preferred choice due to its more accurate treatment [14].

In this report, we present the case of a 38-year-old male with an uncommon presentation of recurrent ankle sprains and a tender ankle mass, ultimately diagnosed with synovial chondromatosis. We will discuss the imaging findings, treatment approaches, and provide a review of the relevant literature.

## Case presentation

A 38-year-old male was referred to the orthopedic surgery clinic with a 6-month history of recurrent right ankle sprains, along with difficulty and pain while walking. The patient reported self-managing his symptoms using over-the-counter (OTC) nonsteroidal anti-inflammatory drugs (NSAIDs) at home during this period without seeking medical attention. On physical examination, a palpable tender mass was found on the anterior aspect of the ankle, with a restricted range of motion in dorsiflexion and mildly limited plantar flexion. No swelling or redness was noted, and the neurological examination of the limb was normal. The patient had no history of trauma, medical conditions, or any significant family or drug history.

**Fig. 1:**
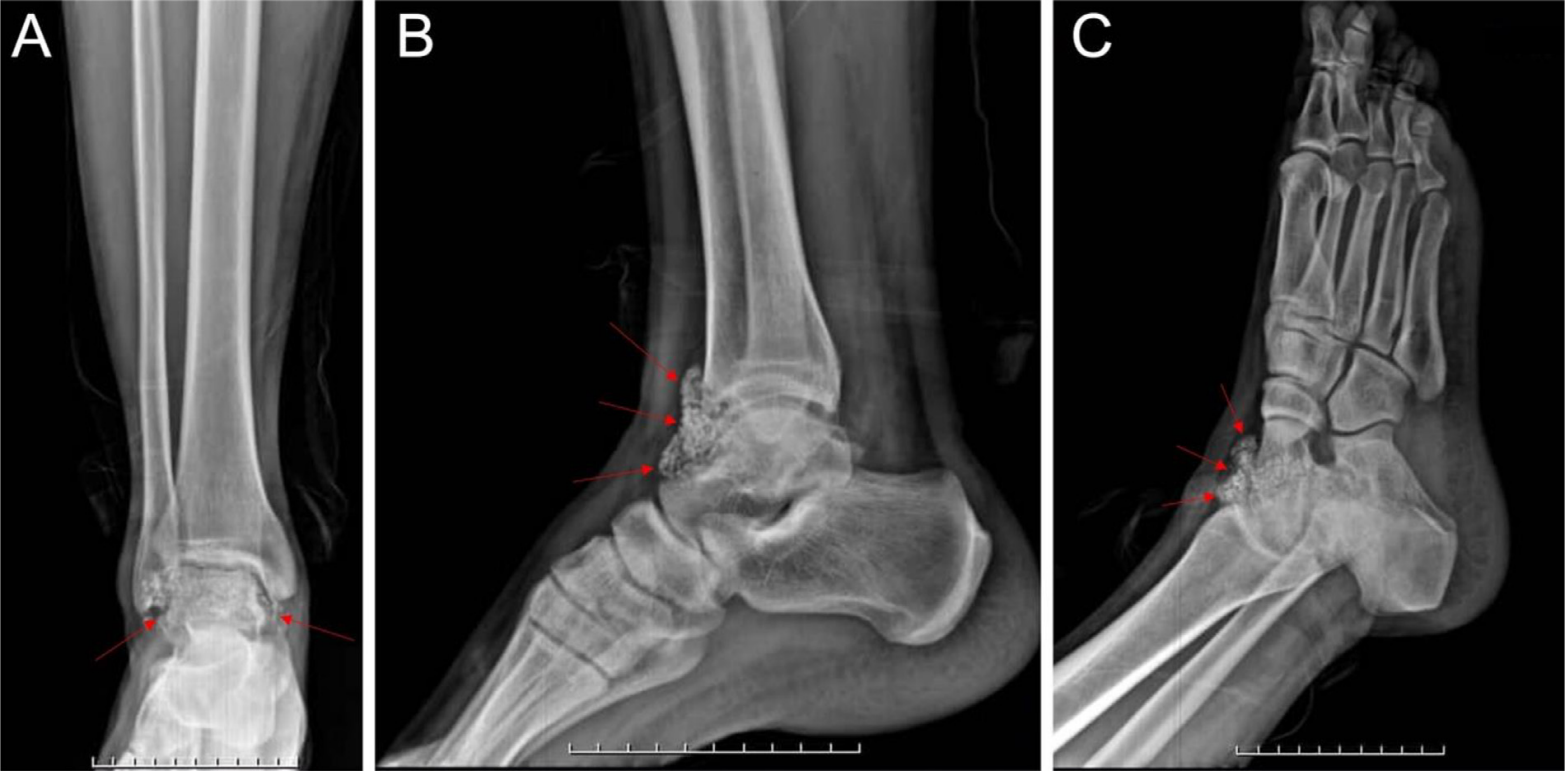
Preoperative plain radiographs of the right foot and ankle showing calcified synovial chondromatosis lesions (loose bodies) in the anterior part of the ankle, indicated by red arrows. Loose bodies, which appear as discrete calcified densities, are seen in A) Anteroposterior (AP) view, (B) Lateral view, and C) Oblique view.

A plain radiograph of the right ankle revealed multiple calcified loose bodies in the anterior aspect of the tibiotalar and tibiofibular joints, consistent with synovial chondromatosis (Fig. 1).

**Fig. 2:**
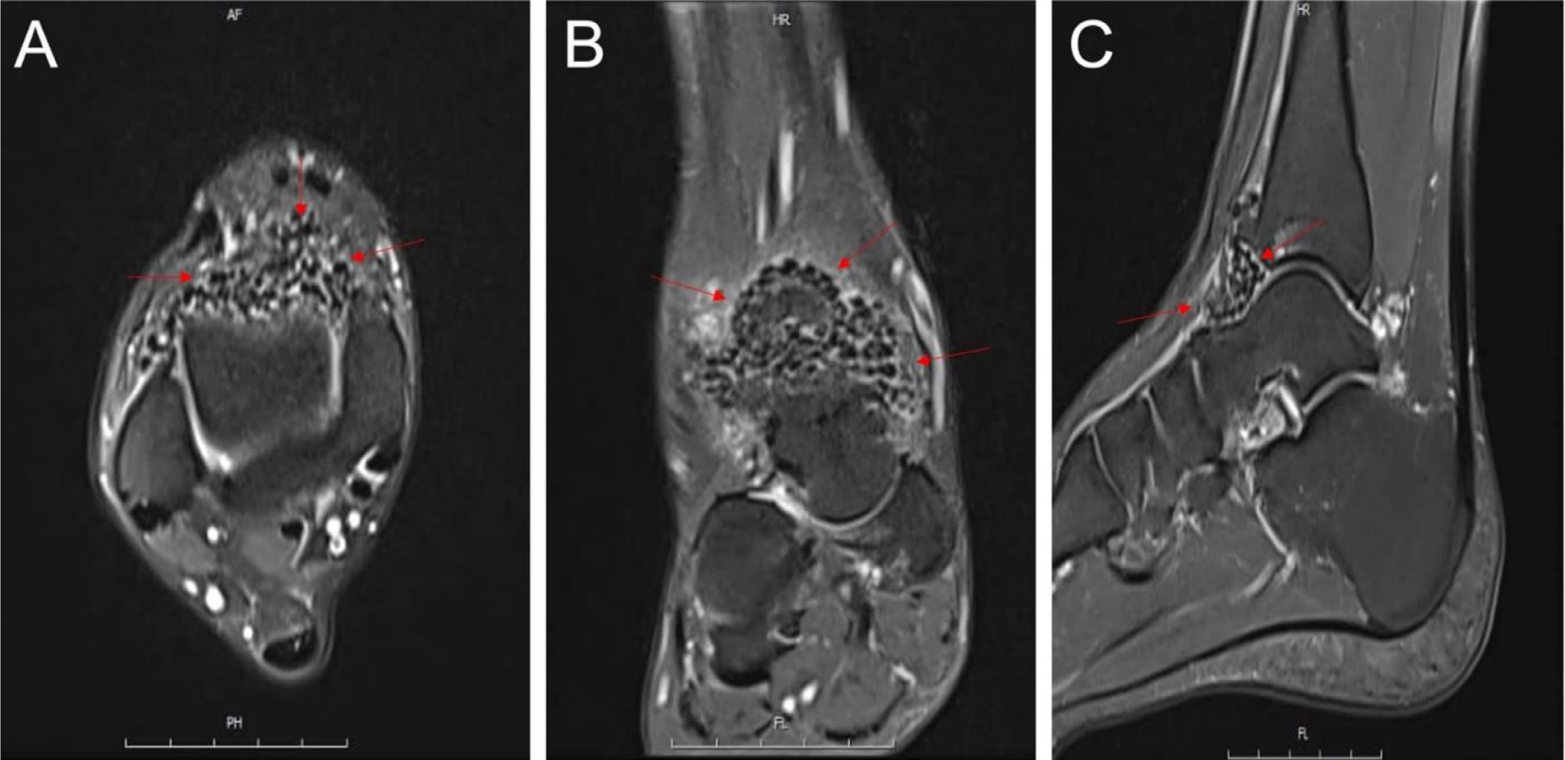
Preoperative MRI of the right foot and ankle showing synovial chondromatosis lesions (loose bodies) in the anterior part of the ankle, indicated by red arrows. The loose bodies appear as low-signal intensity structures within the joint space on MRI, consistent with calcified or ossified tissue. (A) Axial view, (B) Coronal view, (C) Sagittal view. These findings represent intra-articular fragments characteristic of synovial chondromatosis.

For further evaluation, a Magnetic resonance imaging (MRI) of the ankle and foot was performed, which confirmed the diagnosis of synovial chondromatosis (Fig. 2).

The patient was considered for surgical intervention to remove the loose bodies. Initially, ankle arthroscopy was performed, but due to the large number of lesions and limited joint space, the procedure was insufficient. As a result, a second surgery was required, and the patient underwent an open arthrotomy through an anterior midline approach. This allowed for the successful removal of the loose bodies and abnormal tissue, providing a more effective treatment for the synovial chondromatosis.

Multiple extracted lesions were sent for pathological examination, and the diagnosis of primary synovial chondromatosis was confirmed, with no evidence of malignancy detected.

## Patient outcome

**Fig. 3:**
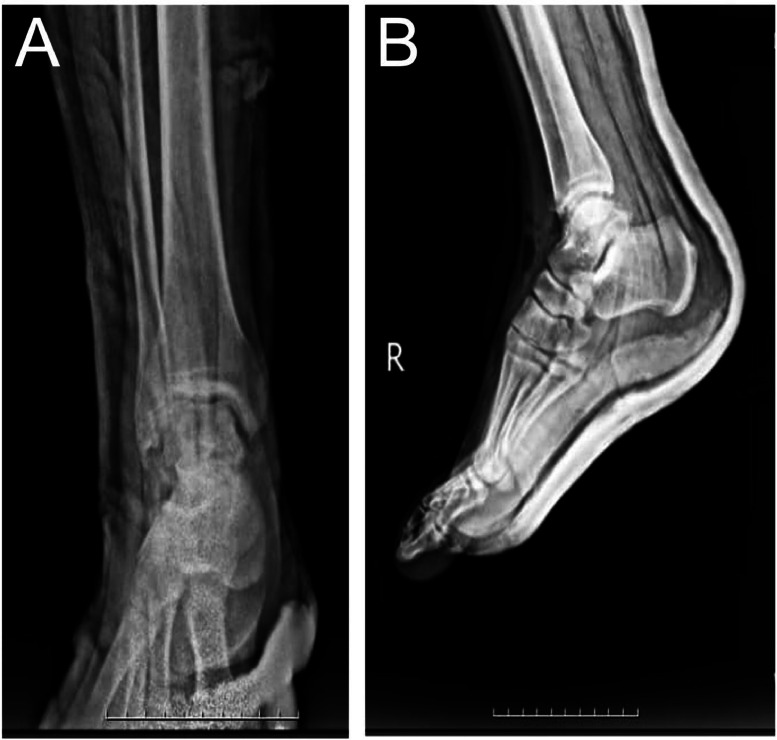
Postoperative plain radiographs of the right foot and ankle 1 week after surgical removal of loose bodies. No residual calcified synovial chondromatosis lesions are observed. (A) Anteroposterior (AP) view, (B) Lateral view. The images demonstrate a clear joint space without evidence of remaining intra-articular fragments.

Follow-up plain radiographs showed a successful outcome (Fig. 3), with all loose bodies fully extracted, and the patient began mobilization 4 weeks after surgery. Since then, all follow-up sessions have been uneventful.

**Fig. 4:**
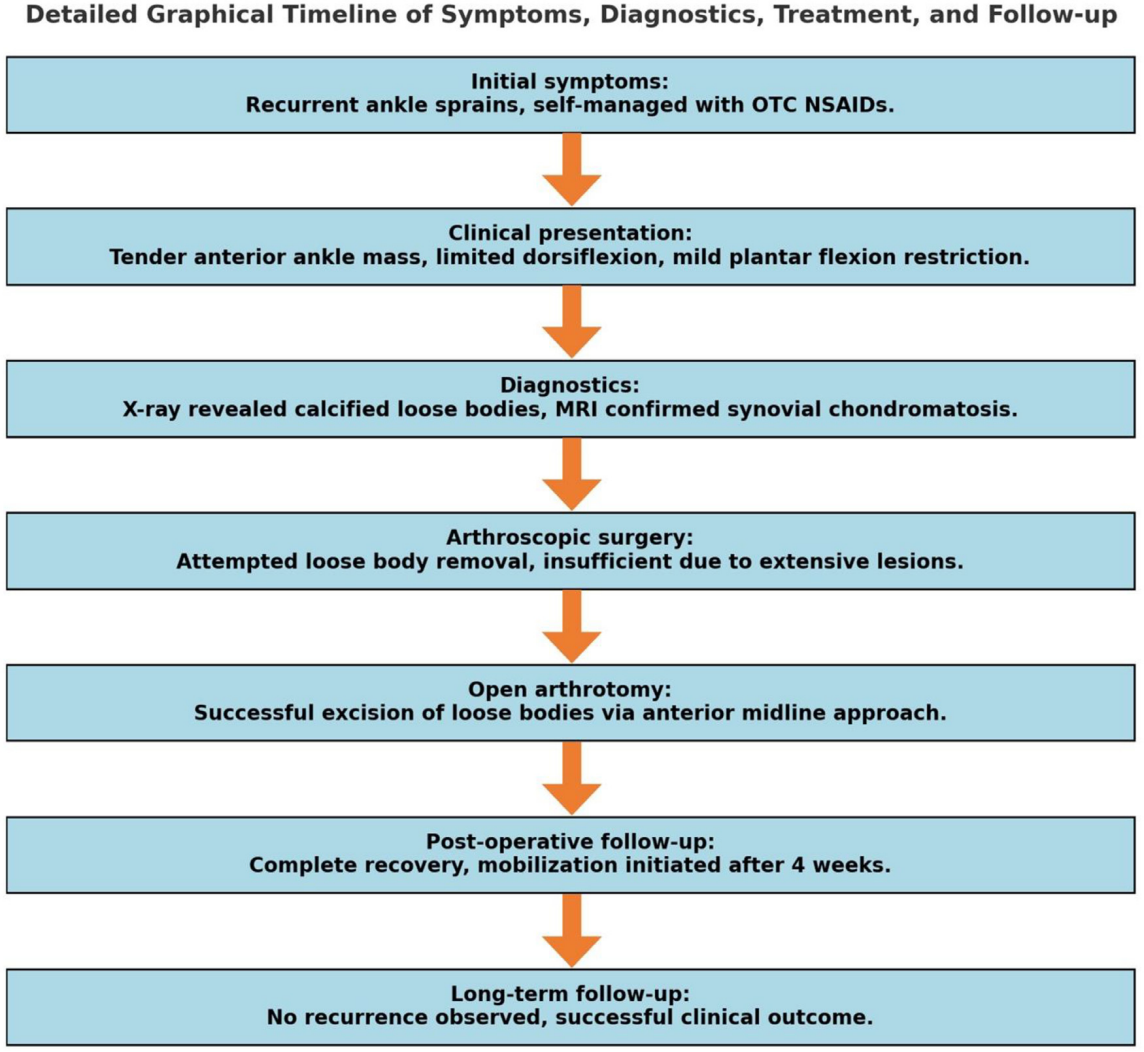
Comprehensive timeline of the patient's clinical presentation, diagnostic findings, surgical interventions, and follow-up outcomes.

The detailed timeline of clinical events and interventions is illustrated in (Fig. 4).

## Discussion

Synovial chondromatosis of the ankle joint is exceptionally rare, with only a few documented cases in medical literature. Notably, 1 study reports a case involving a 9-year-old boy, underscoring the rarity of this condition in pediatric populations [15]. Another case describes a 33-year-old male, further highlighting its infrequency in adults [16]. Generally, synovial chondromatosis is more commonly associated with larger joints such as the knee and hip, making its occurrence in the ankle an outlier [17]. Demographically, this condition typically presents between the ages of 30 and 50 years and is more prevalent in males. However, ankle involvement often occurs in younger patients and does not follow the typical age distribution, as seen in the mentioned pediatric and adult cases [15,18]. The prevalence of synovial chondromatosis is significantly higher in other joints: the knee accounts for 60%-70% of cases, followed by the shoulder, elbow, and hip. The ankle joint, in contrast, has very few documented instances [19,20].

The symptoms of synovial chondromatosis often include pain, swelling, and limited range of motion in the affected joint, along with mechanical symptoms like locking or catching sensations [21,22]. Patients may experience these symptoms for years before diagnosis. Imaging techniques are essential in diagnosing synovial chondromatosis. X-rays, often the initial imaging modality, may show soft tissue swelling and calcified loose bodies but are not always definitive, especially in early stages [23,24]. Magnetic Resonance Imaging (MRI) is particularly useful for visualizing soft tissue structures and provides detailed information about the synovium and loose bodies [23,25]. Computed Tomography (CT) scans can help identify calcifications within loose bodies and assess joint involvement, while high-frequency ultrasound can reveal the presence of nodules or masses [26]. A definitive diagnosis is often confirmed through surgical intervention, where excised tissue is examined histopathologically, revealing cartilaginous nodules characteristic of synovial chondromatosis [22].

Arthroscopic and open surgical techniques are both viable options for addressing joint conditions, but they differ significantly in approach, effectiveness, and recovery outcomes. Arthroscopic techniques are minimally invasive and offer several advantages, including reduced postoperative pain, quicker recovery times, and better visualization of joint structures with minimal soft tissue disruption [27]. However, arthroscopy has limitations when addressing larger loose bodies (>2 cm) or extensive synovial involvement, often making it insufficient for more complex cases [28]. In contrast, open surgical techniques provide direct access to the joint, allowing for the complete removal of large loose bodies and a thorough synovectomy, making it more suitable for cases with significant disease or joint deformity [29]. Despite its effectiveness, open surgery involves larger incisions, longer recovery periods, and a higher risk of complications, such as infection and prolonged rehabilitation [27]. Open arthrotomy is typically chosen when large or numerous loose bodies are present, or when arthroscopic attempts have failed to provide complete excision [28,29]. Treatment guidelines recommend surgical intervention based on the size and number of loose bodies, the extent of synovial proliferation, and the affected joint. While both techniques can achieve favorable outcomes with postoperative rehabilitation, open surgery is often more effective in preventing recurrence, particularly when complete synovectomy is performed [29,30]. Arthroscopic procedures, though beneficial for smaller lesions, may carry a higher recurrence risk due to incomplete removal [30]. Thus, the choice between arthroscopic and open techniques should be based on the complexity of the joint pathology and the desired surgical outcome. This patient's recurrent ankle sprains over 6 months, along with difficulty walking, likely resulted from the mechanical disruption caused by the loose bodies within the joint. The anterior ankle mass and restricted range of motion, particularly in dorsiflexion and plantar flexion, are common findings in advanced disease due to the accumulation of loose bodies that physically limit joint movement. The MRI in this patient confirmed the diagnosis, showing the extent of synovial involvement and the presence of loose bodies. The patient had a successful outcome following the open arthrotomy, with postoperative radiographs confirming the complete removal of the loose bodies. The absence of complications during follow-up and the ability to begin mobilization 4 weeks postsurgery indicate a favorable prognosis. Recurrence is a potential concern in synovial chondromatosis, with rates varying depending on the extent of disease and completeness of excision. Long-term follow-up is thus recommended to monitor for any signs of recurrence.

## Conclusion

This case highlights the importance of considering synovial chondromatosis in patients presenting with chronic joint pain, mechanical symptoms, and recurrent joint instability, particularly in the absence of trauma or underlying joint disease. Early diagnosis and appropriate surgical intervention are key to preventing joint damage and ensuring a favorable outcome. While minimally invasive techniques such as arthroscopy may be effective in less extensive disease, open arthrotomy remains a valuable option for cases with significant involvement, as demonstrated in this patient. Regular follow-up is essential to monitor for recurrence and ensure long-term joint function.

## Patient consent

Written informed consent was obtained from the patient for publication and any accompanying images. A copy of the written consent is available for review by the Editor-in-Chief of this journal on request
